# Seroconverting Blood Donors as a Resource for Characterising and Optimising Recent Infection Testing Algorithms for Incidence Estimation

**DOI:** 10.1371/journal.pone.0020027

**Published:** 2011-06-09

**Authors:** Reshma Kassanjee, Alex Welte, Thomas A. McWalter, Sheila M. Keating, Marion Vermeulen, Susan L. Stramer, Michael P. Busch

**Affiliations:** 1 South African DST/NRF Centre for Epidemiological Modelling and Analysis (SACEMA), University of Stellenbosch, Stellenbosch, South Africa; 2 School of Computational and Applied Mathematics, University of the Witwatersrand, Johannesburg, South Africa; 3 Blood Systems Research Institute, San Francisco, California, United States of America; 4 South African National Blood Service, Johannesburg, South Africa; Scientific Support Office, American Red Cross, Gaithersburg, Maryland, United States of America; University of Cape Town, South Africa

## Abstract

**Introduction:**

Biomarker-based cross-sectional incidence estimation requires a Recent Infection Testing Algorithm (RITA) with an adequately large mean recency duration, to achieve reasonable survey counts, and a low false-recent rate, to minimise exposure to further bias and imprecision. Estimating these characteristics requires specimens from individuals with well-known seroconversion dates or confirmed long-standing infection. Specimens with well-known seroconversion dates are typically rare and precious, presenting a bottleneck in the development of RITAs.

**Methods:**

The mean recency duration and a ‘false-recent rate’ are estimated from data on seroconverting blood donors. Within an idealised model for the dynamics of false-recent results, blood donor specimens were used to characterise RITAs by a new method that maximises the likelihood of cohort-level recency classifications, rather than modelling individual sojourn times in recency.

**Results:**

For a range of assumptions about the false-recent results (0% to 20% of biomarker response curves failing to reach the threshold distinguishing test-recent and test-non-recent infection), the mean recency duration of the Vironostika-LS ranged from 154 (95% CI: 96–231) to 274 (95% CI: 234–313) days in the South African donor population (n = 282), and from 145 (95% CI: 67–226) to 252 (95% CI: 194–308) days in the American donor population (n = 106). The significance of gender and clade on performance was rejected (p−value = 10%), and utility in incidence estimation appeared comparable to that of a BED-like RITA. Assessment of the Vitros-LS (n = 108) suggested potentially high false-recent rates.

**Discussion:**

The new method facilitates RITA characterisation using widely available specimens that were previously overlooked, at the cost of possible artefacts. While accuracy and precision are insufficient to provide estimates suitable for incidence surveillance, a low-cost approach for preliminary assessments of new RITAs has been demonstrated. The Vironostika-LS and Vitros-LS warrant further analysis to provide greater precision of estimates.

## Introduction

Incidence (the rate of new infections) provides a more direct and current indication of the spread of the Human Immunodeficiency Virus (HIV) than prevalence (the fraction of the population in an infected state). Incidence estimates are key to monitoring epidemics, assessing outbreaks, and targeting and evaluating interventions. Prospective longitudinal studies, which allow for the direct counting of new infections in cohorts of individuals, are costly, logistically difficult to set up and maintain, and prone to capturing unrepresentative behaviours. Consequently, estimation of incidence using cross-sectional surveys [1]–[3] has attracted much interest over recent years.

Recent Infection Testing Algorithms (RITAs), often referred to as Serologic Testing Algorithms for Recent HIV Seroconversion (STARHS) [2], classify infections as recently or non-recently acquired. Incidence is then related to the prevalence of RITA-defined recent infection [1]–[11] as estimated in a cross-sectional survey.

RITAs traditionally employ the laboratory measurement of HIV viral or host biomarkers which evolve with time after infection. Antibody avidity, titre, or HIV-specific proportion is typically considered, with a measurement below a chosen threshold indicative of recent infection [12]–[14].

Immune responses vary for individuals, with each individual experiencing a unique evolution of the biomarker. There are two performance characteristics which determine a RITA's utility in population-level incidence estimation.

1. The RITA-defined state of recent infection should not be too transient. This ensures that the proportion of the population in this state may be estimated with good statistical power in surveys with feasible sample sizes. Therefore, the average time spent in the state of recent infection, termed the **mean recency duration**, *ω*, should be large (typically, at least six months [15]).
2. For many RITAs, there is evidence that some long-infected individuals are classified as recently infected [12], [13]. Although the phenomenon of false-recency may, in principle, be accounted for without introducing bias, adjustments result in considerable loss of statistical precision of incidence estimates [15]. The proportion of long-standing infections classified by the RITA as recent, termed the **false-recent rate**, *ε*, should therefore be as low as feasible.

Increasing the threshold (the biomarker cutoff used to discriminate recent from non-recent infection) increases the mean recency duration, but generally also results in a higher false-recent rate. Therefore, as the threshold varies, there is a trade-off between the two performance characteristics. Since population-level surveillance is of interest, rather than each individual's diagnosis, a sensitivity-specificity trade-off (with recent infection defined by a fixed duration) is not an appropriate threshold optimisation criterion (Kassanjee et al, working paper, 2011).

Both calibration data and cross-sectional survey data are required to estimate incidence. Calibration data is used to estimate the RITA characteristics, namely the mean recency duration, *ω*, and false-recent rate, *ε*. Cross-sectional data is used to estimate the proportions of recently infected, non-recently infected and healthy individuals in the population, denoted by *P*_R_, *P*_NR_ and *P*_H_ respectively.

The incidence estimator, relating the population proportions and RITA characteristics to incidence, *I*, is
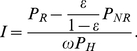
(1)


This has been derived in an analysis by McWalter and Welte [8], shown to be the maximum likelihood estimator by Wang and Lagokos [9], and generalised by Welte et al [15]. See McWalter and Welte [16] for a comparison of this estimator to the previously proposed estimators of McDougal et al [5] and Hargrove et al [6].

Ideally, the RITA should perform similarly in a number of populations, allowing for the reuse of RITA characteristic estimates. However, differences in the stage of the epidemic, or viral subtype or clade, may necessitate the estimation of these critical parameters in relevant populations for each study. For example, the proportions of individuals who are elite controllers (whose immune systems successfully suppress viraemia in the absence of treatment), have advanced immunodeficiency or are receiving antiretroviral therapy may vary, and these individuals have a propensity to produce false-recent classifications [12], [13].

Traditionally, methods of estimating the mean recency duration have relied on the testing of serial samples from acutely infected subjects [1]–[6], [9], [13], [17]. This typically requires at least one pre-seroconversion and multiple post-seroconversion samples, with short intervals between follow-up so that the seroconversion and threshold-crossing times may be estimated with minimal uncertainty. Such panels of data are costly and difficult to capture, requiring precisely the demanding longitudinal studies that cross-sectional incidence estimation seeks to circumvent.

Despite being more easily obtained, specimens from seroconverting subjects with relatively long intervals between follow-up have been largely overlooked. Obtaining such specimens from repeat blood donors provides unique efficiencies as the collection of blood for transfusions is ongoing in most countries, and therefore procuring specimens does not require the establishment of new surveillance. Although the prevalence and incidence of HIV are generally lower in blood donors than the general population, the large-scale collection of blood and routine testing of serial donations for HIV (RNA and antibodies) provide a relatively large sample of seroconverting donors. Furthermore, large volumes of plasma, derived from routinely prepared frozen plasma components, are obtained.

In this investigation, data captured on seroconverting blood donors in South Africa and the USA is used to demonstrate the characterisation and optimisation of RITAs.

## Methods

### Ethics Statement

The research and the incidence testing were approved by the *University of California, San Francisco* (UCSF); *American Red Cross* (ARC) and *South African National Blood Service* (SANBS) institutional review boards or ethics committees.

### Specimen Collection and RITA Testing

Specimens were collected by the *South African National Blood Service* (SANBS) of South Africa and the *American Red Cross* (ARC) of the USA, and tested by the *Blood Systems Research Institute* (BSRI) of the USA. Repeat donors who were observed to seroconvert were tested (by the RITA) using the specimens collected at the times of the first seropositive donations.

The investigation was performed for the less-sensitive Vironostika assay (Vironostika-LS) [18], the RITA for which more data is available, and thereafter, the currently-used less-sensitive Vitros assay (Vitros-LS) [19] was characterised. These RITAs are both based on ‘less-sensitive’ versions of diagnostic tests that measure antibody titre, a concept introduced by Janssen et al [2]. For each RITA, recent infection is indicated by a standardized optical density (SOD) below a chosen threshold.

The Vironostika-LS is a modification of the Vironostika HIV-1 microELISA diagnostic test (bioMérieux, Marcy l'Étoile, France) [18]. The laboratory procedures and threshold of 1 specified by Rawal et al [18] were used. Seroconverting blood donors were tested using the Vironostika-LS until 2007, as production of the Vironostika assay ceased in the year thereafter [13]. Manufacturing of the assay has since been resumed by Avioq (Rockville, MD) [20].

The Vitros-LS is based on the Ortho Vitros ECi anti-HIV 1+2 instrument (Ortho-Clinical Diagnostics, Raritan, NJ) [19]. The BSRI established the laboratory conditions that result in the closest agreement to classifications by the Vironostika-LS, using a threshold of 20 [19].

The datasets consist of the SOD at the time of the first seropositive donation, and the interval between the last seronegative (and RNA negative) and first seropositive donation, termed the inter-donation (ID) interval, for each seroconverting blood donor. Three datasets (Supplemental Digital Content (SDC) S1, Section A) were used for the analysis: The Vironostika-LS was applied to samples of South African donors (October 2005 - September 2007, sample size of *n* = 485) and North American donors (November 2001 - December 2005, *n* = 176); the Vitros-LS was applied to a sample of South African donors (October 2007 – December 2009, *n* = 199).

### Analysis

RITA characteristics were estimated using a maximum likelihood method. Rather than fitting a curve describing the evolution of the SOD with time after seroconversion, the overall probability of the RITA classifications at the first seropositive donations in the sample was maximized [21]. The likelihood function is derived below (more detail provided in SDC S1, Section B).

Assuming that the time of seroconversion is uniformly distributed in the ID interval, the probability, *p_i_*, that the *i* ^th^ seroconverter, with ID interval Δ*_i_*, is classified as recently infected at the time of the first seropositive donation is
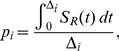
(2)


where *S_R_(t)* is the probability of being in the RITA-defined state of recent infection when tested a time *t* after seroconversion.

The likelihood, *L*, to be maximised, of all RITA classifications in a sample of *n* seroconverters is
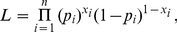
(3)


where *x_i_* = {1 if RITA-recent, 0 if RITA-non-recent} is the observed result for the *i* ^th^ seroconverter.

The analyses of McDougal et al [5], McWalter and Welte [8] and Wang and Lagakos [9] assume that individual SOD curves either cross the threshold (distinguishing test-recent from test-non-recent infection) and remain above it or fail to ever reach the threshold, and therefore *S_R_(t)* in (2) approaches some constant value, α, which is the proportion of SOD curves that fail to reach the threshold, for large *t*. *S_R_(t)* may then be expressed as
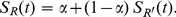
(4)


The mean recency duration, *ω*, is the mean of the times taken to cross the threshold for those SOD curves that do cross the threshold, as described by *S_R'_(t).*

Substituting (4) into (2), *p_i_* becomes
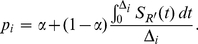
(5)


This approach also facilitates non-parametric inference, by considering only individuals with large Δ*_i_*. For a time cutoff *T* such that
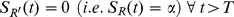
(6)


if Δ_i_>*T*, then
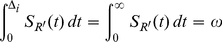
(7)


is the mean recency duration.

Substituting (7) into (5), *p_i_* becomes a function of the RITA characteristics,
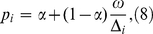
(8)


and no assumptions about the shape with which the biomarker grows after seroconversion (full characterisation of *S_R'_(t)*) is required. The estimated RITA characteristics maximise the likelihood *L*, which is now a function of *ω*, and *α* (if there is no input estimate of α).

McDougal et al [5], McWalter and Welte [8] and Wang and Lagakos [9] additionally assume that post-seroconversion survival is independent of the shape of the SOD curves. When the above-mentioned assumptions are obeyed, *α = ε* in the incidence estimator (1). More generally, *S_R_(t)* may not remain constant for *t*>*T*. A false-recent rate may then be defined as the proportion of individuals, seropositive for longer than *T*, that is classified as recently infected [15]. In this case, the above procedure that produces an estimate of *α* likely overestimates the false-recent rate if SOD curves cross the threshold after *T* or underestimates it if SOD curves move back below the threshold at times since seroconversion greater than that captured in the dataset. The estimated RITA characteristics, *α* and *ω*, therefore provide unrefined estimates for the false-recent rate and mean recency duration.

Uniformly distributed seroconversion times are reasonable when the timing of donations and exposures to HIV are independent. Test-seeking behaviour (the donation of blood soon after exposure specifically to receive HIV testing) or deferral of donations (the delay of donations soon after exposure) could therefore bias estimates. In the USA, an investigation, which highlighted test-seeking behaviour among homosexual men, noted little indication of test-seeking behaviour among blood donors [22], while evidence of deferred donations has been observed [23]. Behaviour in the South African donor population may vary due to the large scale of the epidemic and stigma associated with HIV.

In this work, various analyses involving the parametric and non-parametric inference of the RITA characteristics for the Vironostika-LS and Vitros-LS were performed. Parametric inference was performed by maximising the likelihood function based on the probability of being recently infected expressed in (5), assuming forms for *S_R'_(t)* and using all data; non-parametric inference was performed using a likelihood function based on (8) and only including data satisfying Δ_i_>*T*. Using simulated data, estimates obtained from the parametric and non-parametric approaches were compared. Differences in RITA characteristics for specific subpopulations were explored. The utility of the RITA for obtaining precise incidence estimates was also investigated.

Asymptotic maximum likelihood theory was used to estimate confidence intervals (CIs) and confidence regions (CRs), and test the significance of parameters (based on the distribution of the deviance statistic and using the loglikelihood ratio test) [24]. Chi-squared goodness of fit tests were used to assess the agreement between data and assumptions [25]. All tests used a significance level of 5%.

## Results

### Characterisation of the Vironostika-LS

**Figure 1:**
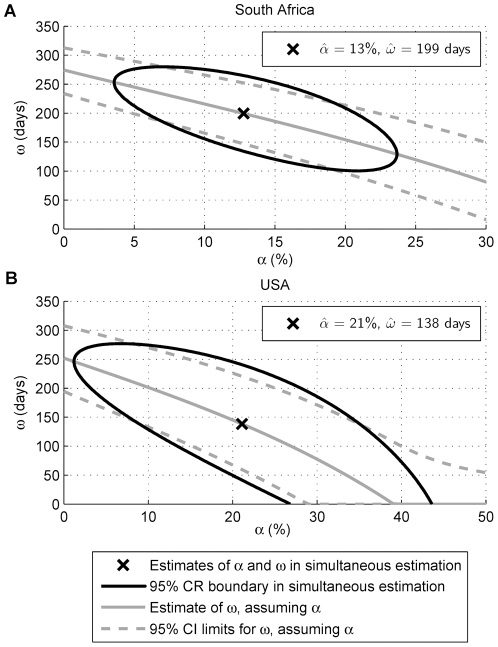
Estimated RITA characteristics for the Vironostika-LS in the repeat donor population. The estimates of the mean recency duration, *ω*, under both the simultaneous estimation of *ω* and *α*, and when using an input *α*, for *T* = 1 year, are provided. For the latter estimation, the estimated *ω* is plotted as a function of the assumed *α*. The 95% confidence regions (CRs) for *ω* and *α* (simultaneous estimation) and confidence intervals (CIs) for *ω* (assuming α, and not accounting for uncertainty in α) are displayed. Part A shows the results for the South African repeat donor sample, while Part B shows the results for the USA repeat donor sample.

The estimated RITA characteristics (using a threshold of 1) are shown (Fig. 1), for both estimation assuming a known *α*, and simultaneous estimation of *ω* and *α*. Observations with Δ_i_>*T* = 1 year were used (the maximum duration in the state of recency has been estimated to be 200 days [26] and 1 year [17]), resulting in sample sizes of *n* = 282 and *n* = 106 for the South African and American datasets respectively. A comparison of the observed percentages of seroconverters who were recently infected to the expected percentages (by substituting the estimated RITA characteristics into (8)), as functions of ID interval, suggests good agreement under simultaneous estimation of the RITA characteristics (SDC S1, Section C). When exploring the sensitivity of results to *T*, when *T* was increased to 2.5 years, estimates from the South African dataset varied by at most 10% (*n* = 189), while the large uncertainty in estimates from the relatively small American dataset (*n* = 53) did not support meaningful inference.

The estimated α is large, consistent with the results from the application of this method to assess the BED assay [21(results not shown)]. Estimation of *ω* using an input estimate of *α* is preferable. In the extreme case of all ID intervals being equal, *ω* and *α* cannot be simultaneously estimated as the likelihood function may be kept at its maximum while arbitrarily increasing the estimate of *ω* by appropriately decreasing the estimate of *α*. Furthermore, using a value of *T* that is too low (SOD curves cross the threshold after *T*) would bias estimates of *α* upwards and *ω* downwards under the assumptions of McDougal et al [5], McWalter and Welte [8] and Wang and Lagakos [9], with larger *T* required at higher thresholds.

**Figure 2:**
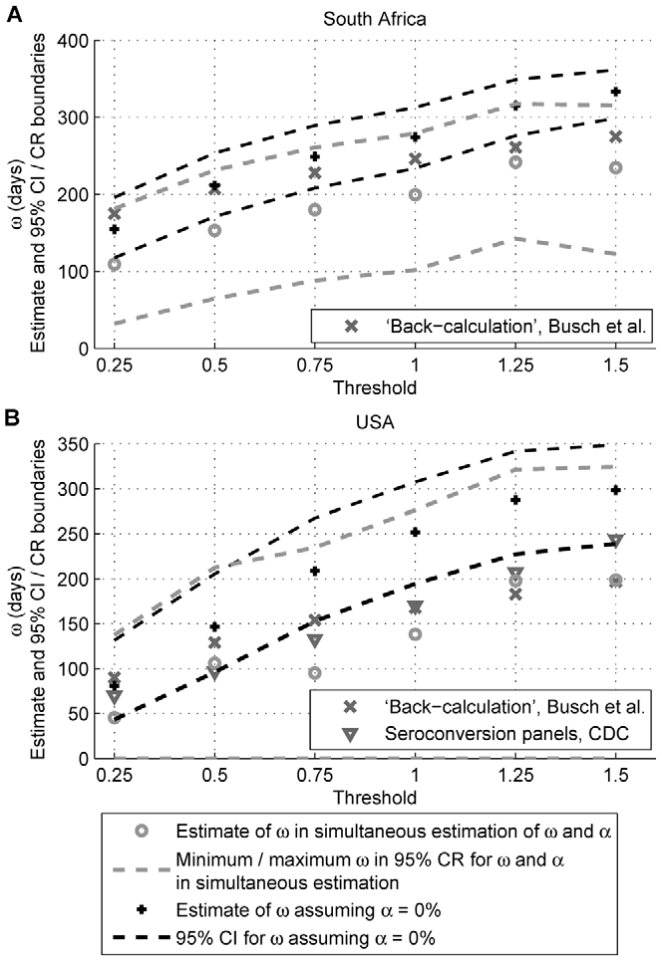
Comparison of mean recency duration estimates for the Vironostika-LS to previously published estimates. Estimates of the mean recency duration, *ω*, under both the simultaneous estimation of *ω* and *α*, and when assuming *α* = 0%, for *T* = 1 year, are compared to published estimates (by ‘back-calculation’ in the repeat donor population [27] and using seroconversion panels [18], [27]) as a function of test threshold. The minimum and maximum *ω* occurring in the 95% confidence regions (CRs) for *ω* and *α* (simultaneous estimation), as well as 95% confidence interval (CI) limits for *ω* (assuming *α* = 0%, with no uncertainty) are also displayed. Estimates shown in Part A pertain to the South African population, while those in Part B pertain to the USA population.

The estimated mean recency durations, for a number of thresholds (holding *T* at 1 year), are compared to published estimates (Fig. 2):

1. Busch et al [27] utilised the directly measured incidence in the repeat donor population to estimate *ω*. With known incidence; proportions *P*_R_, *P*_NR_ and *P*_H_ (determined by testing repeat donors); and assuming *ε* = 0%; a ‘back-calculation’ for *ω* using the incidence estimator was performed. Since the possibility of false-recent results was neglected, overestimation of *ω* is expected, with greater bias at higher thresholds. Methodologically, estimation of *ω* by ‘back-calculation’ requires an existing estimate of *ε* for the same threshold, with such data currently unavailable. Furthermore, uncertainty in the estimate of *ω* arises from uncertainty in the estimated incidence; proportions *P*_R_, *P*_NR_ and *P*_H_; and *ε*.
2. The *Centers for Disease Control and Prevention* (CDC) utilised seroconversion panels to estimate *ω* in an American population [18], [27].

### Parametric versus non-parametric approach

The need for parametric assumptions about the shape of the antibody titre response curve is circumvented by using only data with large ID intervals. This has two consequences:

1. Estimation of *ω* is less prone to bias arising from poor parametric assumptions.
2. The dataset used for the estimation is reduced in size, decreasing the precision (increasing the variability) of estimates of *ω*.

The characterisation of the Vironostika-LS in the South African repeat donor population was revisited, using all data. The probability that a seroconverter is recently infected at the first seropositive donation is given by (5), which assumes a parametric form for *S_R'_(t)*.

For *S_R'_(t) = S_R'_(θ,t)*, where *θ* is a vector of parameters, the maximum likelihood estimator of *ω* is
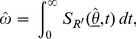
(9)


where *θ* is estimated to maximise the likelihood function.

A number of forms for *S_R'_(t)* were assumed, ranging from a fixed recency duration for all individuals, to a fat-tailed Pareto distribution (for the time spent in the state of recency). Widely varying estimates of *ω* (SDC S1, Section D) were obtained, even after excluding estimates for which assumptions and data poorly agreed. Since the true underlying dynamics of the data are unknown, the extent of bias is unclear.

Simulated data was therefore used to explore the trade-off between greater precision from larger samples and greater potential for bias from poor parametric assumptions, when using all data. Assuming a number of forms for *S_R'_(t)*, 100 datasets (of 500 seroconverters each) were simulated. For each dataset, *ω* was estimated using a number of parametric assumptions. The results of the investigation (SDC S1, Section D) suggest that power to reject ‘incorrect’ parametric assumptions is at times poor and large bias in estimates may occur. When the assumptions leading to (8) hold, estimates using the non-parametric approach are unbiased, although less precise.

Despite the reduction in sample size (approximately 40% for *T* = 1 year) when using the non-parametric method of estimation, bias arising from indistinguishably poor parametric assumptions is eliminated, leading to more accurate estimates.

### Population-specific performance

Significant systematic bias could be introduced to incidence estimates if the RITA characteristics are not evaluated in a population representative of that in which incidence estimation is to occur [11], [15]. Since most HIV antibody assays are based primarily on clade B antigens, antibody-antigen reactivity may vary when applying assays in populations in which other clades occur [28], with differences in the performance of the Vironostika-LS already observed [18], [26], [28]–[30]. Other factors, such as the association between viral RNA levels and clade, and seroconverters’ genetic backgrounds, may also affect results [4], [12], [13], [28].

The significance of gender (male and female) and country (South Africa and USA) on the performance of the Vironostika-LS was assessed. Country differences are likely to be largely representative of clade differences, as clade C infections are predominant in South Africa, and clade B in the USA [31]. Investigations by SANBS on a sample of donors (data made available to authors) and studies of North American donors [32], [33] indicate that a very small percentage (<5%) of infections are not of the predominant clade.

**Figure 3:**
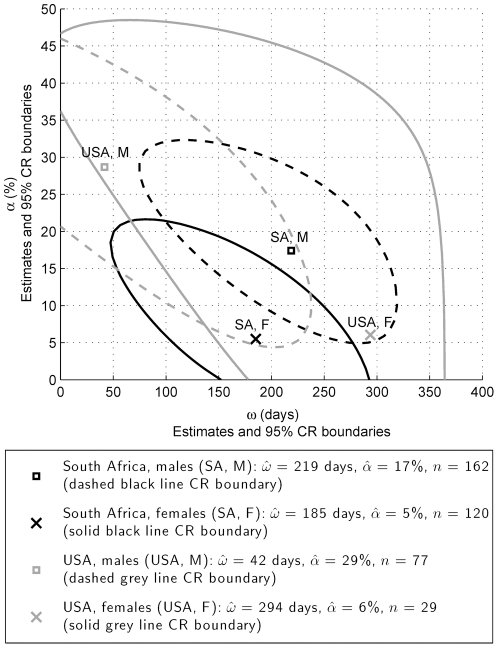
Estimated RITA characteristics for the Vironostika-LS in the repeat donor population by gender and country. Estimates of *ω* and *α*, under the simultaneous estimation of these parameters, are shown for South African male donors, South African female donors, USA male donors and USA female donors. 95% confidence regions (CRs) for *ω* and *α* are provided.

The null hypothesis, that the performance of the Vironostika-LS is common in all four groups (each pairing of gender and country), is not rejected with a p-value of 10.48% (estimated RITA characteristics in Fig. 3). However, in this investigation, large uncertainty in estimates, arising from small samples of seroconverters, would result in little power to identify significant factors.

### Optimisation of RITA design and comparison of RITAs

**Figure 4:**
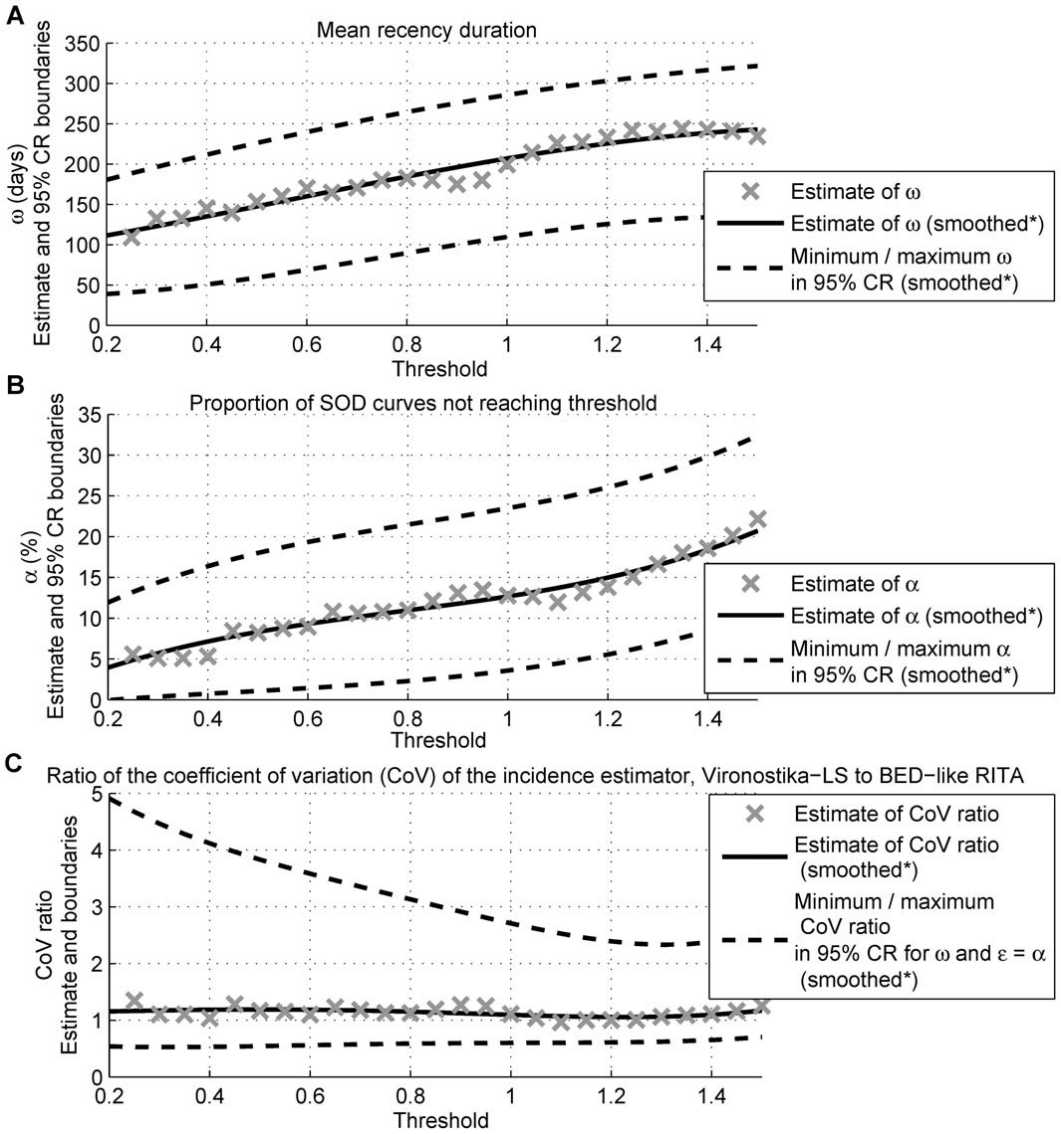
Performance of the Vironostika-LS for incidence estimation, based on estimated RITA characteristics. The estimated performance of the Vironostika-LS, for incidence estimation purposes, is shown, based on estimated RITA characteristics for the South African repeat donor population. In Part A and Part B, estimates of *ω* and *α*, respectively, under the simultaneous estimation of these parameters, for *T* = 1 year, are plotted as a function of test threshold. The minimum and maximum *ω* and *α* occurring in the 95% confidence regions (CRs) for these parameters are also displayed. In Part C, the estimated precision of the incidence estimator using the Vironostika-LS is compared to the precision obtained by a BED-like RITA (*ω* = 155 days and *ε* = 5.6% [34]), based on the estimated RITA characteristics (assuming *ε* = *α*) and assuming constant HIV incidence of 1.5% and prevalence of 17.5%. More specifically, the ratio of the coefficient of variation (CoV, ratio of standard deviation to mean) of the incidence estimator, for the Vironostika-LS to the BED-like RITA, is plotted as a function of the Vironostika-LS test threshold. *A polynomial is fitted by least squares to smooth estimates.

The ultimate objective is incidence estimation. The precision of the incidence estimator (and hence power to detect changes in incidence) increases with a larger mean recency duration and smaller false-recent rate [15]. However, there is a fundamental trade-off between these RITA characteristics as both parameters increase with increasing threshold, shown for the Vironostika-LS, South Africa (Fig. 4A and Fig. 4B, *α* provides an indication of the magnitude of the false-recent rate).

The coefficient of variation (CoV, ratio of standard deviation to mean) of the incidence estimator [8], given the estimated performance of the Vironostika-LS, is compared to that achieved by a BED-like RITA (*ω* =  days and *ε*  = 5.6%, as per BED package insert [34]), assuming *ε* = *α* (Fig. 4C). HIV incidence of 1.5% and prevalence of 17.5% are assumed, based on the South African adult population [35], [36]. With the CoV ratio (Vironostika-LS to BED-like) indistinguishable from 1, at all thresholds considered, the Vironostika-LS appears comparable to a BED-like RITA. Additional data, such as captured during the follow-up of seropositive individuals awaiting treatment, could be used to explore whether systematic artefacts in the estimation occur (for example, from individuals progressing after *T* = 1 year).

### Characterisation of the Vitros-LS

**Figure 5:**
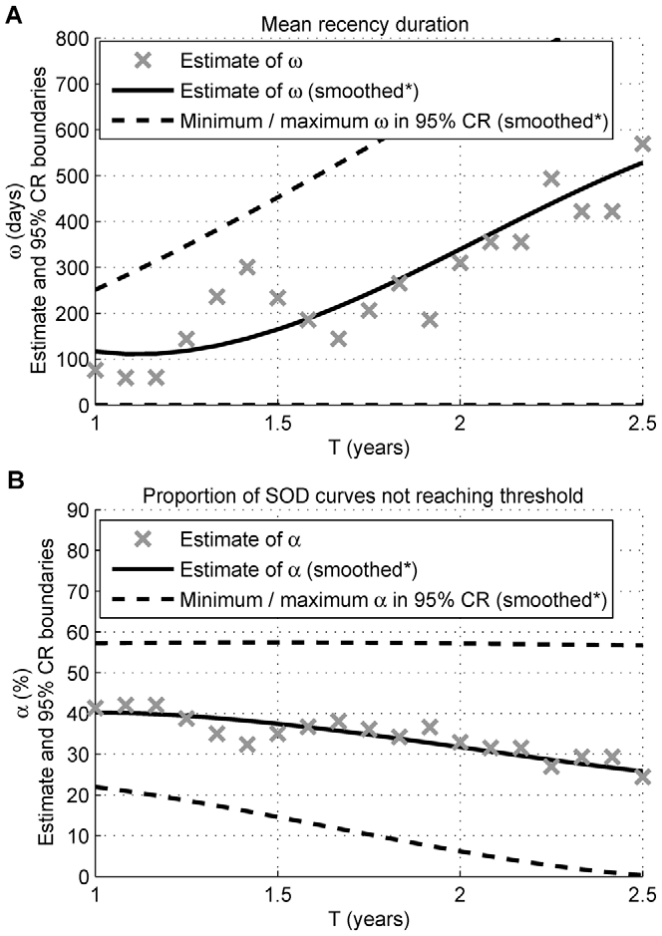
Estimated RITA characteristics for the Vitros-LS in the South African repeat donor population. The estimates of the RITA characteristics, *ω* and *α*, under the simultaneous estimation of the parameters are provided, as a function of the cutoff time *T,* for *T* = 1 year to *T* = 2.5 years. In Part A and Part B, estimates of *ω* and *α* are plotted, respectively. The minimum and maximum *ω* and *α* occurring in the 95% confidence regions (CRs) for these parameters are also displayed. *A polynomial is fitted by least squares to smooth estimates.

Preliminary RITA characteristic estimates of the currently used Vitros-LS (using a threshold of 20), for the South African repeat donor population, are shown (Fig. 5). The simultaneous estimation of *ω* and *α*, for a range of *T*, was performed (*n* = 108 for *T* = 1 year reduces to *n* = 59 for *T* = 2.5 years). Observed and expected percentages of seroconverters who were recently infected were also compared (SDC S1, Section E).

For the Vitros-LS, the RITA characteristics were plotted as a function of cutoff time *T* (Fig. 5) as no estimates for *T* were found in the literature and the estimation appeared fairly sensitive to the choice of *T* for this dataset. The widely varying estimates for the RITA characteristics, and large uncertainty around them, indicate the need for a larger dataset and an external estimate of *α* for a carefully selected cutoff time *T* (large enough for the SOD curves to cross the threshold) to get a more accurate and precise estimate of the mean recency duration. Such data and estimates of *α* and *T* are currently unavailable. While a value of *T* that is too small would bias estimates of *α* upwards and *ω* downwards, under the above-mentioned assumptions, as the value of *T* increases, the sample size reduces and interdonation intervals are more closely clustered together, decreasing the power to perform simultaneous estimation.

The large, albeit highly uncertain, estimates of *α* suggest that one should be cautious about the utility of the Vitros-LS in incidence estimation at this stage of the characterisation, noting that *α* is not the false-recent rate in (1) for *S_R_(t)* not (approximately) constant for *t>T*.

## Discussion

Traditionally, the characterisation of RITAs (individual assays and multiple-test algorithms) has relied on the use of seroconversion panels. The scarcity of these panels is therefore an obstacle to the development of RITAs for incidence estimation. In this work, a source of more readily available specimens has been identified, and an approach for obtaining preliminary characterisations of RITAs using these specimens has been demonstrated. Further refinement of the characterisation of only the most promising RITAs may thereafter be performed, thus conserving precious longitudinal specimens (and specimens from populations with long-standing infections) for this purpose.

Utilising specimens from blood donors provides unique efficiencies as relatively large samples of seroconverters and high-volume specimens (125-250 ml of plasma per seroconverter) are captured during routine blood collection procedures. Furthermore, specimens from seropositive individuals around the world are collected, facilitating tests for population-specific performance differences of a RITA.

The method of estimating the RITA characteristics (mean recency duration and a proxy ‘false-recent rate’ for parameter estimation purposes) does not require the follow-up of seroconverters. Moreover, by using data with large (pre-seroconversion) follow-up intervals, non-parametric estimation is supported. To obtain more accurate and precise estimates of the mean recency duration, an external estimate of the proportion of SOD curves that does not reach the threshold is desirable, as well as insight into the maximum time seroconverters spend in the test-recent state.

For incidence estimation, the utility of the Vironostika-LS appears comparable to a BED-like RITA, over the range of thresholds considered. The precision of the incidence estimator provides a criterion for both comparing RITAs as well as identifying optimal thresholds. While additional data is required for the Vitros-LS, preliminary results suggest prudence when utilising the assay for incidence estimation.

The assumptions under which estimates are unbiased are strict. Potential for systematic bias in estimates, such as that arising from individuals remaining in the state of recency for prolonged periods, or from non-uniformly distributed seroconversion times, should be explored using additional data. This method of estimating the RITA characteristics is not intended to provide final parameter estimates required for incidence estimation, but rather for providing cost-effective and efficient preliminary assessments of RITAs. It is hoped that the concepts and tools demonstrated in this work will contribute to the resourceful characterisation, and subsequently focused development, of RITAs for population-level incidence estimation.

## Supporting Information

Supplemental Digital Content (SDC) S1 (PDF)
