# Supplementary material for: Seroconverting Blood Donors as a Resource for Characterising and Optimising Recent Infection Testing Algorithms for Incidence Estimation
Source: PLoS One. 2011 Jun 9;6(6):e20027. doi: 10.1371/journal.pone.0020027 (PMC3111407; doi:10.1371/journal.pone.0020027)
Supplement: Supplemental Digital Content (SDC) S1 — (PDF) [file pone.0020027.s001.pdf]

Seroconverting Blood Donors as a Resource for Characterising and Optimising Recent Infection Testing Algorithms for Incidence Estimation:

Supplemental Digital Content

Reshma Kassanjee[1,2], Alex Welte[1], Thomas A McWalter[1,2], Sheila M Keating[3], Marion Vermeulen[4], Susan L Stramer[5] and Michael P Busch[3]

[1] South African DST/NRF Centre for Epidemiological Modelling and Analysis (SACEMA), University of Stellenbosch, Stellenbosch, South Africa

[2] School of Computational and Applied Mathematics, University of the Witwatersrand, Johannesburg, South Africa

[3] Blood Systems Research Institute, San Francisco, CA USA

[4] South African National Blood Service, Johannesburg, South Africa

[5] American Red Cross, Scientific Support Office, Gaithersburg, MD USA

This Supplemental Digital Content (SDC) provides additional material for the work presented in:

Kassanjee R, Welte A, McWalter TA, Keating SM, Vermeulen M, Stramer SL, Busch MP. Seroconverting Blood Donors as a Resource for Characterising and Optimising Recent Infection Testing Algorithms for Incidence Estimation. *PLoS ONE*, 2011.

A The Data

Scatter plots of the datasets (by Recent Infection Testing Algorithm (RITA) and country of collection) are provided below. The standardised optical density (SOD) at the time of the first seropositive donation and the interdonation (ID) interval between the last seronegative and first seropositive donation, for each seronverting blood donor, are shown. SOD values below the thresholds (indicated by blue lines) indicate recent infections.

Figure 1: Vironostika-LS, South Africa

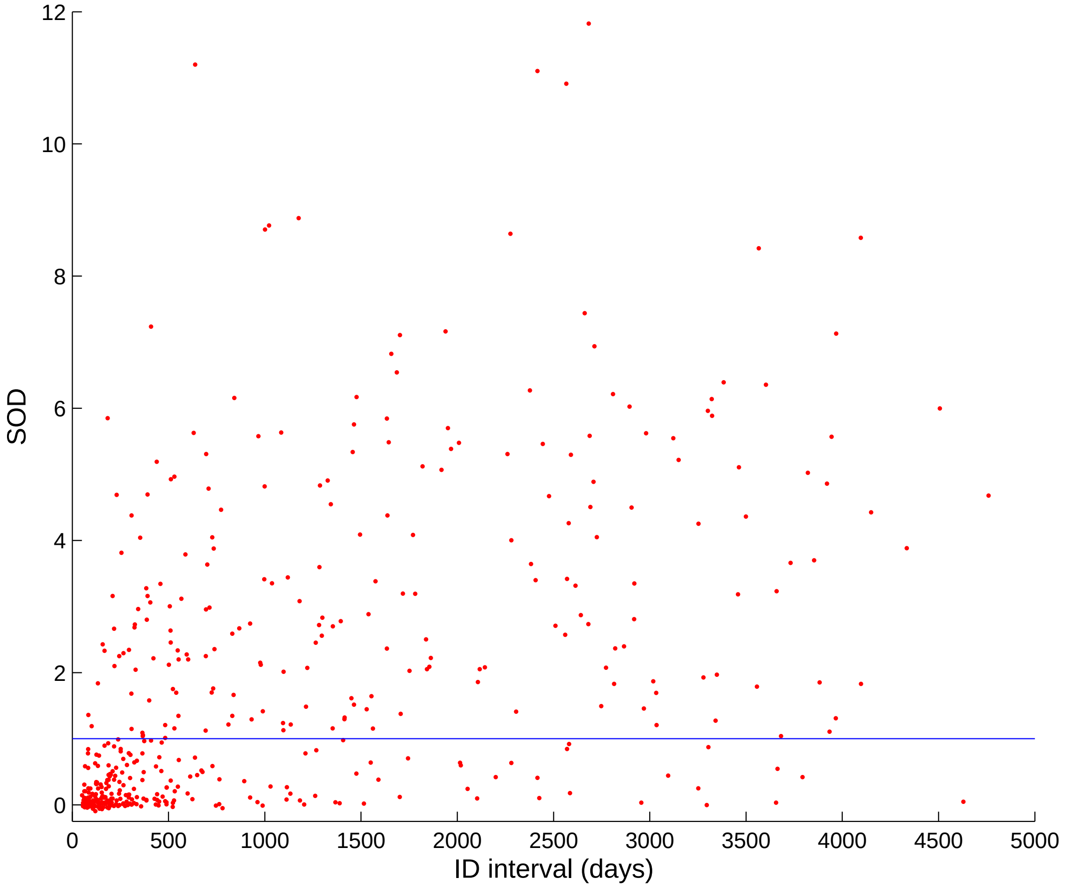

Figure 2: Vironostika-LS, USA

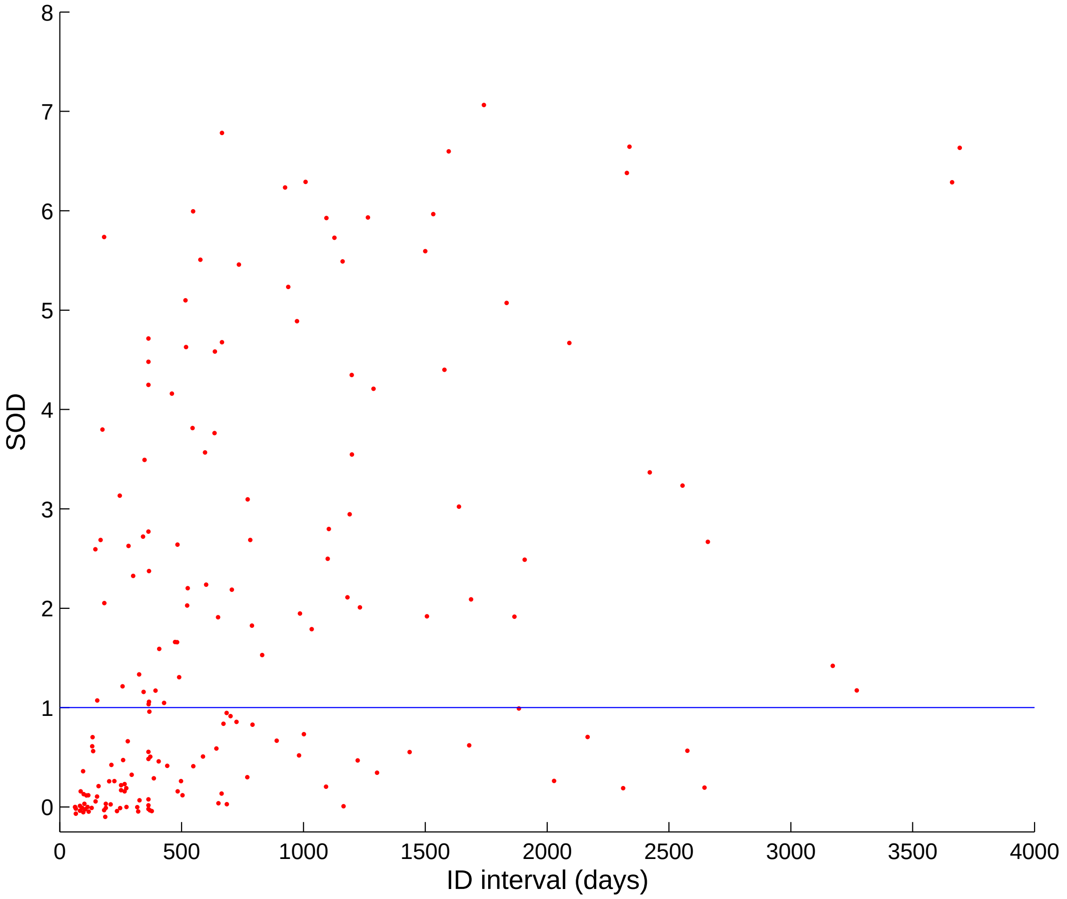

Figure 3: Vitros-LS, South Africa

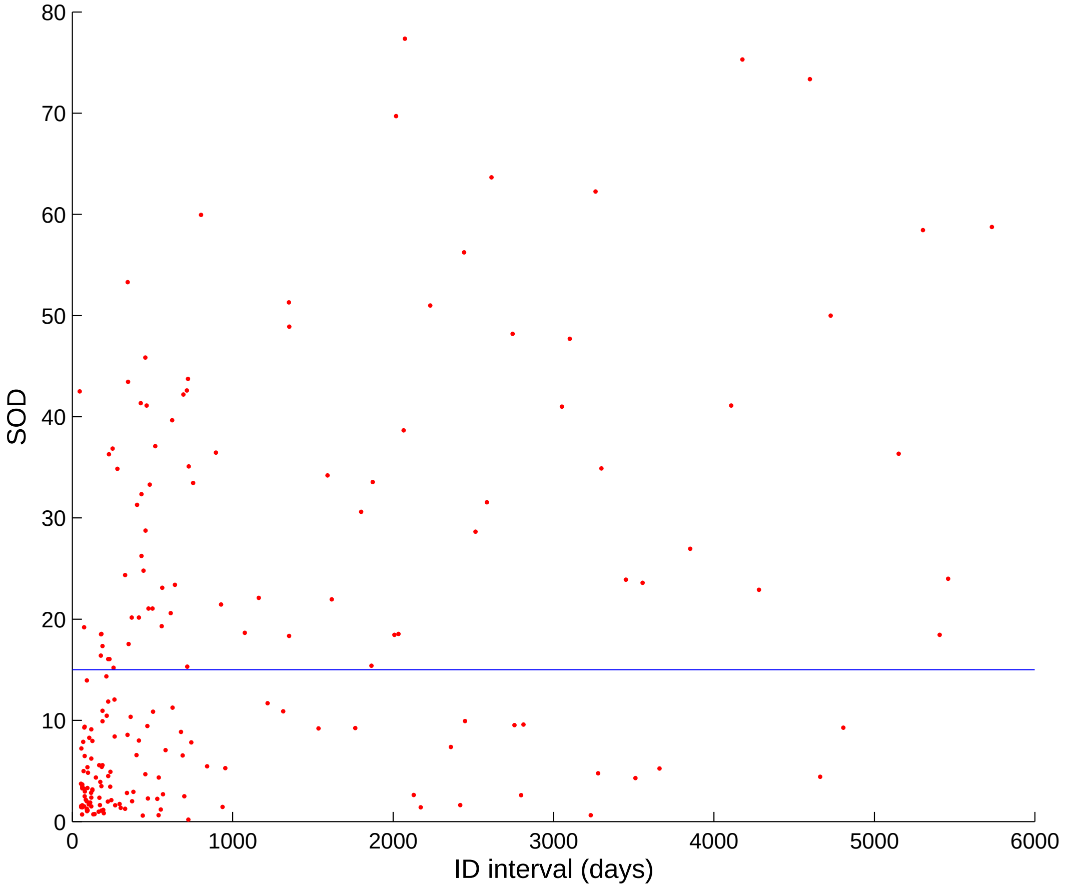

B The RITA Characteristic Estimators

The maximum likelihood estimators for the RITA characteristics to be estimated are derived below. The distributional properties of the estimators, for large samples, are also noted.

Derivation of the Maximum Likelihood Estimators

For a seroconverter with interdonation (ID) interval Δ between the last seronegative test and first seropositive test:

1. X denotes the result of the RITA at the time of the first seropositive test, and has a probability mass function $f_X(x)$,

$$X = \begin{cases} 1 & \text{if recently infected} \\ 0 & \text{if non-recently infected} \end{cases}.$$

2. Y is the time since seroconversion at the time of the first seropositive donation, and the time of seroconversion is uniformly distributed in the ID interval,

$$f_Y(y) = \frac{1}{\Delta} \quad 0 \leq y \leq \Delta.$$

   The joint probability function of X and Y is denoted by $f_{X,Y}(x,y)$, and the distribution of X conditional on Y by $f_{X|Y}(x|y)$.

3. $S_{\mathrm{R}}(t)$ is the probability that the seroconverter is in the state of recent infection a time t after seroconversion, conditional on being alive. $S_{\mathrm{R}}(t) = f_{X|Y}(x|y)$.

The probability, p, that the seroconverter is classified as recently infected at the time of the first seropositive donation is

$$\begin{aligned} p &= f_X(1) \\ &= \int_0^{\Delta} f_{X,Y}(1,t)\,\mathrm{d}t \\ &= \int_0^{\Delta} f_Y(t)\, f_{X|Y}(1|t)\,\mathrm{d}t \\ &= \int_0^{\Delta} \frac{1}{\Delta} S_{\mathrm{R}}(t)\,\mathrm{d}t \\ &= \frac{\int_0^{\Delta} S_{\mathrm{R}}(t)\,\mathrm{d}t}{\Delta}. \end{aligned} \tag{1}$$

The likelihood, L, of all RITA classifications in a sample of n seroconverters is

$$L = \prod_{i=1}^{n} (p_i)^{x_i} \, (1 - p_i)^{1-x_i} \,, \tag{2}$$

where the subscript i denotes quantities relating to the ith seroconverter in the sample, and x denotes the observed values of X.

The analyses of McDougal et al [1], McWalter and Welte [2] and Wang and Lagakos [3] assume individual SOD curves either cross the threshold (distinguishing recent from non-recent infection) and remain above it or fail to reach the threshold, and therefore $S_R(t)$ approaches some constant value, α, which is the proportion of SOD curves that fail to cross the threshold, for t larger than some time cutoff T,

$$S_{\mathrm{R}}(t) = \alpha + (1 - \alpha)\, S_{\mathrm{R}'}(t), \tag{3}$$

The mean recency duration, ω, is the mean of the times taken to cross the threshold, for those SOD curves that do so, described by $S_{\mathrm{R}'}(t)$.

More generally, $S_{\mathrm{R}}(t)$ may not remain constant for $t > T$. A false-recent rate, ε, may then be defined as the proportion of individuals, who have been seropositive for longer than T, that is classified as recently infected [4].

For $S_{\mathrm{R}}(t)$ exhibiting little variability around an approximately constant value for $t > T$, the parameterisation in (3) is used to obtain rough estimates of the RITA characteristics.

Substituting (3) into (1), the probability that the seroconverter is recently infected at the time of the first seropositive donation becomes

$$p = \alpha + (1 - \alpha)\, \frac{\int_0^{\Delta} S_{\mathrm{R}'}(t)\, \mathrm{d}t}{\Delta}. \tag{4}$$

For $S_{\mathrm{R}'}(t) = S_{\mathrm{R}'}(\underline{\theta}, t)$, L is a function of the unknown parameters $\underline{\theta}$ and α (if there is no input estimate for α), which are estimated to maximise L. The estimate of ω is $\int_0^{\infty} S_{\mathrm{R}'}(\hat{\underline{\theta}}, t)\, \mathrm{d}t$, where $\hat{\underline{\theta}}$ is the estimate of $\underline{\theta}$.

This likelihood approach also facilitates non-parametric inference, by considering only individuals with large Δ. Since

$$S_{\mathrm{R}'}(t) = 0 \text{ (i.e. } S_{\mathrm{R}}(t) = \alpha) \qquad \text{for} \qquad t > T, \tag{5}$$

if $\Delta > T$, then

$$\int_0^{\Delta} S_{\mathrm{R}'}(t)\, \mathrm{d}t = \int_0^{\infty} S_{\mathrm{R}'}(t)\, \mathrm{d}t = \omega \tag{6}$$

is the mean recency duration.

Substituting (6) into (4), p becomes a function of the RITA characteristics,

$$p = \alpha + (1 - \alpha)\, \frac{\omega}{\Delta}, \tag{7}$$

and the likelihood function becomes

$$L = \prod_{i=1}^{n^*} (p_i)^{x_i} \, (1 - p_i)^{1-x_i} \qquad \text{where} \qquad p_i = \alpha + (1-\alpha)\,\frac{\omega}{\Delta_i} \tag{8}$$

and $n^*(\leq n)$ is the size of the sample consisting of all seroconverters with ID intervals larger than T (and the subscript i denotes quantities relating to the ith individual in this smaller sample). The estimated RITA characteristics maximize the likelihood L, which is now a function of ω, and α (if there is no input estimate of α).

Simultaneous estimation of the RITA characteristics is less feasible in samples with closely clustered ID intervals. In the extreme case of $\Delta_i = \Delta$ for all i, simultaneous estimation is not possible as there are no unique estimates of ω and α which maximise the likelihood function

$$L \propto \left(\alpha + (1-\alpha)\,\frac{\omega}{\Delta}\right)^{\sum_{i=1}^{n^*} x_i} \left(1 - \alpha - (1-\alpha)\,\frac{\omega}{\Delta}\right)^{\sum_{i=1}^{n^*} (1-x_i)}, \tag{9}$$

(which is maximised when $\dfrac{\sum_{i=1}^{n^*} x_i}{n^*} = \alpha + (1-\alpha)\,\dfrac{\omega}{\Delta}$).

Properties of the Estimators

A maximum likelihood estimator, $\hat{\underline{\xi}}$, is asymptotically (as the sample size $n \to \infty$) normally distributed around the true parameter value, $\underline{\xi}$, with variance equal to the inverse of the expected Fisher's Information Matrix:

$$\hat{\underline{\xi}} \xrightarrow{\mathrm{d}} N\left(\underline{\xi}, \left[-\mathbb{E}\left(\frac{\partial^2 \ln(L(\underline{\xi}))}{\partial \underline{\xi}^2}\right)\right]^{-1}\right) \quad \text{as } n \to \infty \tag{10}$$

where $\mathbb{E}(.)$ is the expected value and $L(.)$ is the likelihood function, under regularity conditions [5].

Large sample approximations for the properties of the estimators for the mean recency duration and proportion of SOD curves that fail to reach the threshold, $\hat{\omega}$ and $\hat{\alpha}$, follow.

1. α **known**

$$\hat{\omega} \sim N\left(\omega, \left[\sum_{i=1}^{n^*}\left(\frac{1-\alpha}{\Delta_i}\right)^2\left(\frac{1}{p_i}+\frac{1}{1-p_i}\right)\right]^{-1}\right), \tag{11}$$

where p_i and $L = L(\omega)$ are given in (8).

2. α **unknown**

$$\begin{bmatrix} \hat{\omega} \\ \hat{\alpha} \end{bmatrix} \sim N\left(\begin{bmatrix} \omega \\ \alpha \end{bmatrix}, \left[-\mathbb{E}\begin{pmatrix} \frac{\partial^2 \ln(L)}{\partial \omega^2} & \frac{\partial^2 \ln(L)}{\partial \omega\, \partial \alpha} \\ \frac{\partial^2 \ln(L)}{\partial \alpha\, \partial \omega} & \frac{\partial^2 \ln(L)}{\partial \alpha^2} \end{pmatrix}\right]^{-1}\right), \tag{12}$$

where the covariance matrix is

$$\begin{bmatrix} \sum_{i=1}^{n^*}\left(\frac{1-\alpha}{\Delta_i}\right)^2\left(\frac{1}{p_i(1-p_i)}\right) & \sum_{i=1}^{n^*}\frac{1}{\Delta_i p_i} \\ \sum_{i=1}^{n^*}\frac{1}{\Delta_i p_i} & \sum_{i=1}^{n^*}\left(1-\frac{\omega}{\Delta_i}\right)^2\left(\frac{1}{p_i(1-p_i)}\right) \end{bmatrix}^{-1}, \tag{13}$$

and p_i and $L = L(\omega, \alpha)$ are given in (8).

C Fit of Estimated RITA Characteristics, Vironostika-LS

The method of maximum likelihood, outlined in Section B above, was used to characterise the Vironostika-LS, in the South African and American repeat donor populations. Firstly, the mean recency duration, ω, was estimated assuming a known α. Secondly, simultaneous estimation of ω and α was performed. Non-parametric estimation was applied, using data on seroconverters with interdonation (ID) intervals larger than $T = 1$ year ($n = 282$ for South Africa and $n = 106$ for USA). In the figures below, the observed percentages and expected percentages (obtained by substituting estimated RITA characteristics into (7)) of seroconverters who were recently infected at the first seropositive donations, as a function of ID interval, are compared. Subjects with similar ID intervals were grouped together (at least 20 subjects per group), and the observed and expected percentages were plotted against the average ID interval, per group. In Figures 4 and 6, the 95% confidence interval limits for the expected percentages (dotted lines) are obtained by substituting the 95% confidence interval limits for ω, not taking any uncertainty in α into account, into (7). In Figures 5 and 7, the plotted limits for the expected percentages (dotted lines) indicate the minimum and maximum values for the probability of being recently infected, p, obtained when considering all pairs of values for the RITA characteristics lying within the 95% confidence regions for ω and α.

Figure 4: Agreement between the observed and expected percentages of recently infected seroconverters for the Vironostika-LS, in the South African repeat donor population, assuming a known α, for $T = 1$ year

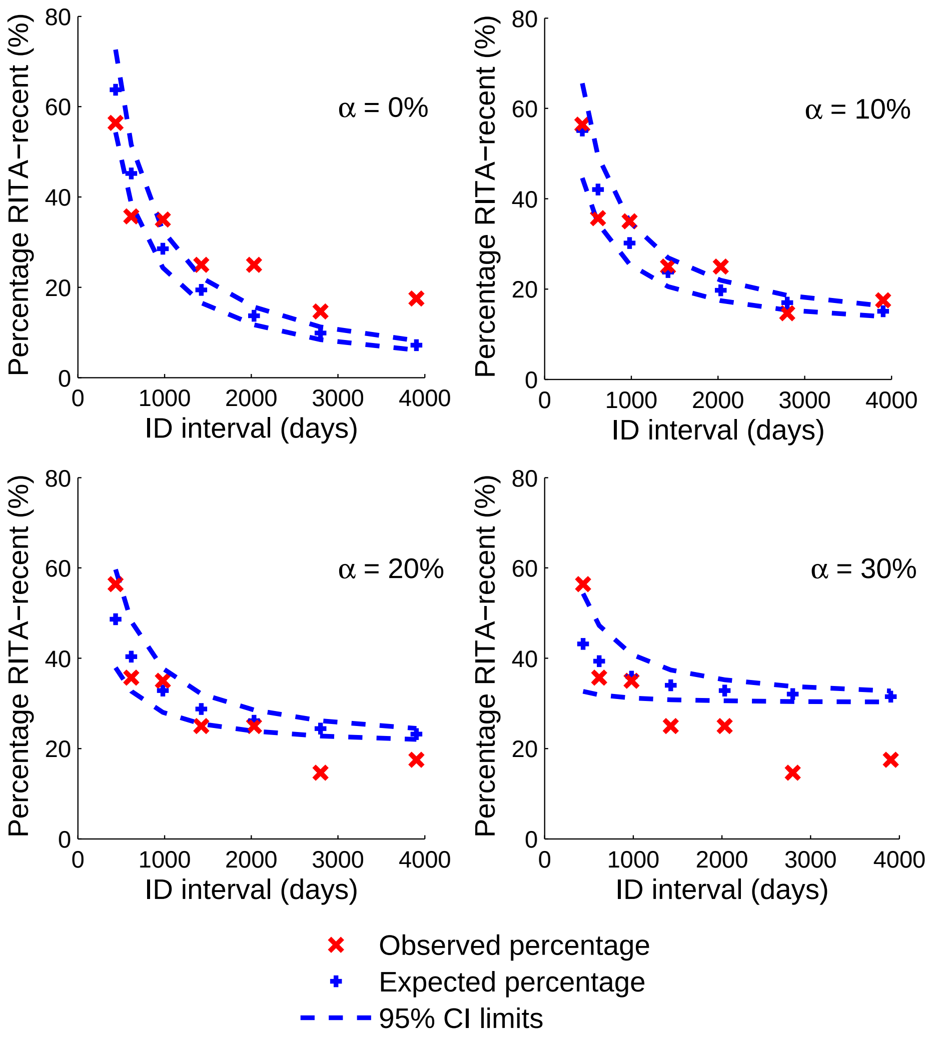

Figure 5: Agreement between the observed and expected percentages of recently infected seroconverters for the Vironostika-LS, in the South African repeat donor population, simultaneously estimating ω and α, for $T = 1$ year

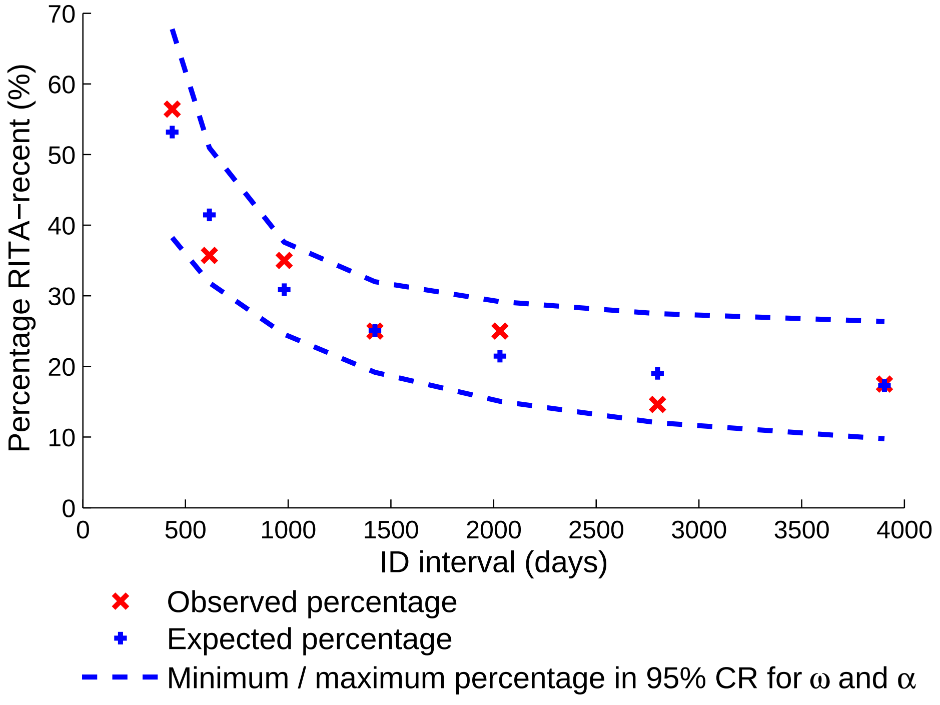

Figure 6: Agreement between the observed and expected percentages of recently infected seroconverters for the Vironostika-LS, in the American repeat donor population, assuming a known α, for $T = 1$ year

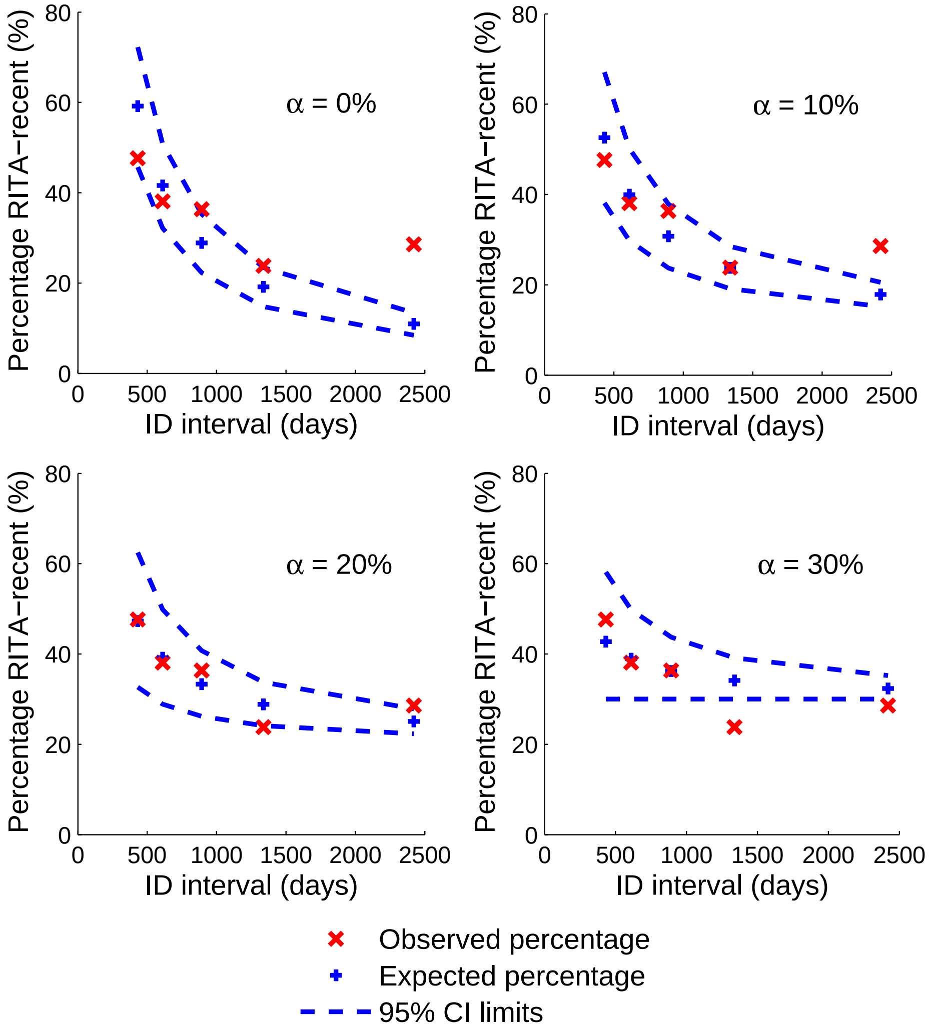

Figure 7: Agreement between the observed and expected percentages of recently infected seroconverters for the Vironostika-LS, in the American repeat donor population, simultaneously estimating ω and α, for $T = 1$ year

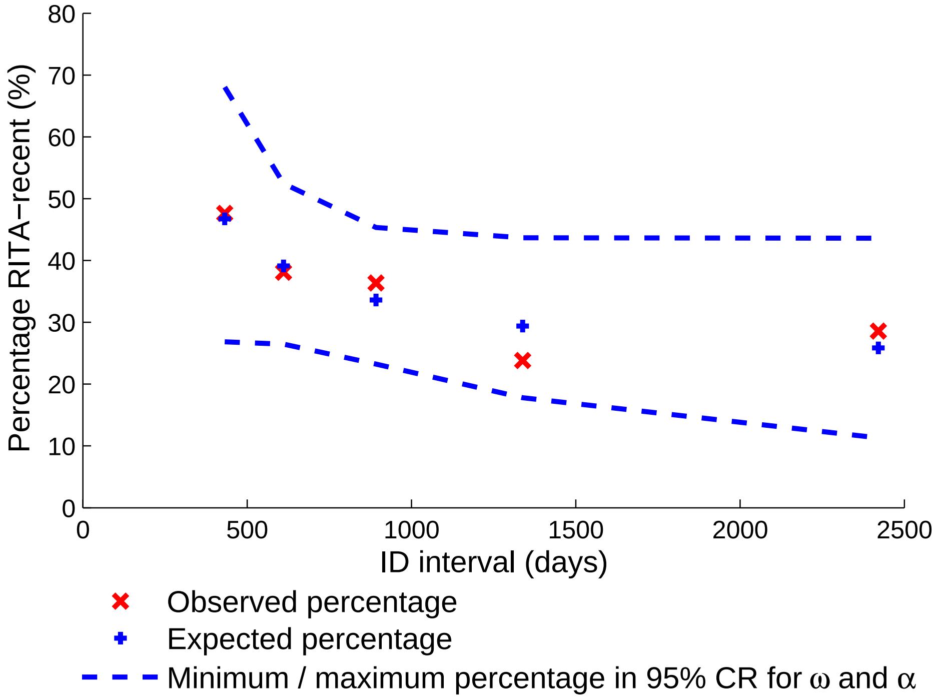

D Parametric Versus Non-Parametric Estimation

By using only data with sufficiently large interdonation (ID) intervals, the need for parametric assumptions is circumvented (see Section B above). While this protects against bias arising from poor parametric assumptions, the sample size is reduced.

The RITA characteristics of the Vironostika-LS, in the South African repeat donor population, were estimated using all data and a number of parametric assumptions (characterisations of $S_{\mathrm{R}'}(t) = S_{\mathrm{R}'}(\underline{\theta}, t)$, where $\underline{\theta}$ is a vector of parameters). The seven assumed forms for $S_{\mathrm{R}'}(\underline{\theta}, t)$ are plotted in Figure 8. By design, $\underline{\theta} = \omega$ for each form.

In Table 1, estimates of the mean recency duration, ω, using the various parametric assumptions, are tabulated. The results of the chi-squared goodness of fit tests [6], used to assess the agreement between the data and assumptions, are also provided. Widely varying estimates of ω were obtained, even after discarding those estimates for which data and assumptions poorly agreed. Since the underlying dynamics of the data are unknown, the extent of bias is unknown.

Simulated data was therefore used to investigate the trade-off between the increased precision from larger samples and increased potential for bias from poor parametric assumptions, when moving to a parametric approach. 100 datasets (of 500 seroconverters each) were simulated, assuming each of the seven forms for $S_{\mathrm{R}'}(\underline{\theta}, t)$ and $\alpha = 0\%$. ID intervals were simulated from a non-parametric distribution fitted to the ID intervals in the dataset for the Vironostika-LS, South Africa. For each dataset, the goodness of fit was assessed and ω estimated, using each parametric assumption. The non-parametric method was also applied, using all ID intervals greater than $T = 1$ year. Underestimation of ω is therefore expected for the distributions with maximum times in the state of recent infection greater than 1 year.

The results of the investigation, provided in Table 2, indicate that, although moving to a parametric approach allows all data to be exploited, there is the potential of introducing large bias in estimates from indistinguishably poor parametric assumptions. The average 95% confidence interval widths, when using the correct parametric assumptions and the non-parametric approach, are also provided in Table 2. The increased widths when moving to the non-parametric approach illustrate the loss of precision incurred when discarding data with insufficiently large ID intervals.

Figure 8: The various parametric assumptions, plotted for a mean recency duration, ω, of 150 days

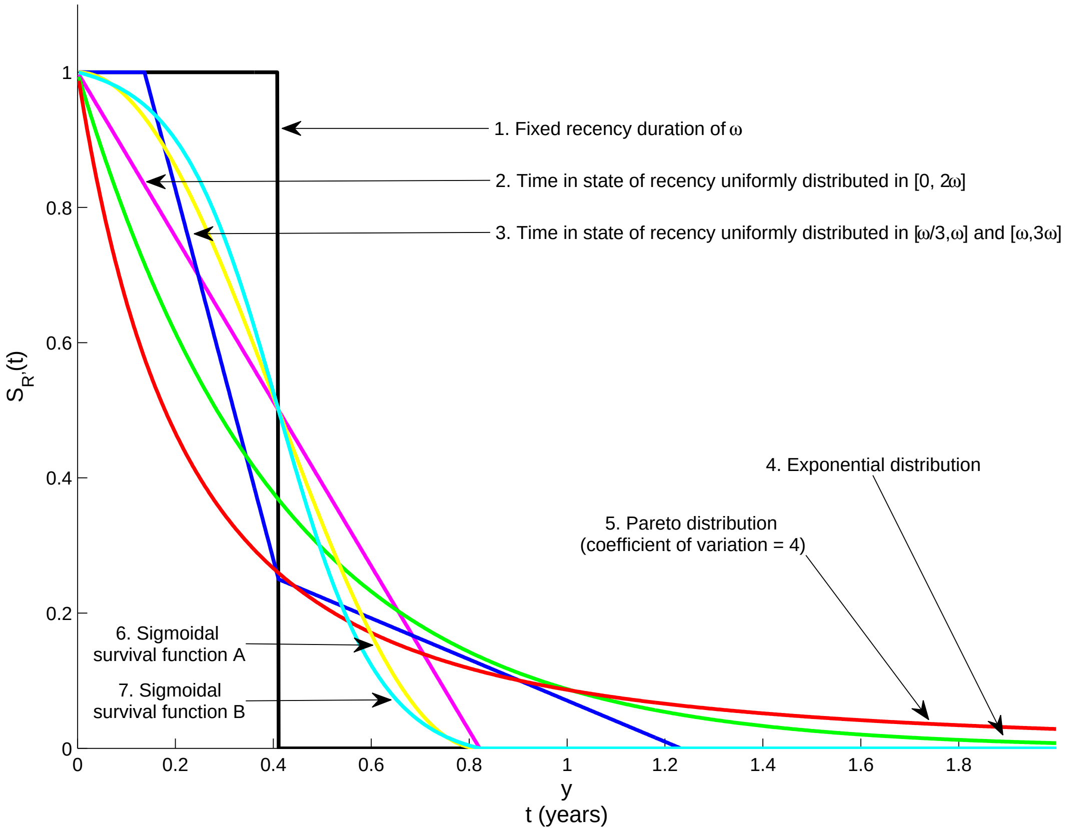

Table 1: Estimated mean recency duration for the Vironostika-LS, in the South African repeat donor population, using various parametric assumptions

		Estimate of mean recency duration, days (95% CI)				Goodness of fit test: Result (p-value)[1]			
		α = 0%	α = 5%	α = 10%	α = 15%	α = 0%	α = 5%	α = 10%	α = 15%
Parametric estimation: Assumed survival function	1	84 (82-84)	83 (81-84)	83 (81-84)	83 (80-84)	Reject (0%)	Reject (0%)	Reject (0%)	Reject (0%)
	2	**316 (266-374)**	**278 (233-333)**	**251 (209-301)**	**229 (188-276)**	**Fail to Reject (29%)**	**Fail to Reject (10%)**	**Fail to Reject (56%)**	**Fail to Reject (73%)**
	3	237 (217-249)	**228 (204-246)**	**219 (192-242)**	**208 (180-235)**	Reject (3%)	**Fail to Reject (57%)**	**Fail to Reject (65%)**	**Fail to Reject (80%)**
	4	429 (355-520)	**379 (309-464)**	**338 (273-418)**	**303 (242-378)**	Reject (2%)	**Fail to Reject (12%)**	**Fail to Reject (16%)**	**Fail to Reject (9%)**
	5	**650 (528-802)**	579 (464-721)	**516 (409-649)**	461 (361-585)	**Fail to Reject (9%)**	Reject (2%)	**Fail to Reject (8%)**	Reject (3%)
	6	**268 (232-309)**	**241 (207-281)**	**221 (188-259)**	**205 (173-242)**	**Fail to Reject (5%)**	**Fail to Reject (56%)**	**Fail to Reject (69%)**	**Fail to Reject (90%)**
	7	**274 (235-318)**	**245 (210-287)**	**225 (190-264)**	**208 (175-246)**	**Fail to Reject (8%)**	**Fail to Reject (73%)**	**Fail to Reject (91%)**	**Fail to Reject (85%)**
Non-parametric estimation		**274 (234-313)**	**245 (199-289)**	**216 (165-266)**	**186 (132-241)**				

[1] Null hypothesis: The data is consistent with the assumed survival function (survival in state of recent infection), significance level of 5%

Table 2: Estimated mean recency duration for the simulated data, using various parametric assumptions, where the true mean recency duration is 150 days

Mean of non-rejected estimated mean recency durations, days (% of estimates rejected) [Average 95% CI width, days]		Parametric estimation: Assumed survival function							Non-parametric estimation
		1	2	3	4	5	6	7	
'True' distribution from which data generated	1	**153 (2%) [27]**	187 (87%)	197 (23%)	- (100%)	- (100%)	174 (28%)	171 (33%)	**150 (0%) [79]**
	2	- (100%)	**153 (2%) [51]**	129 (67%)	191 (7%)	273 (22%)	138 (32%)	142 (12%)	**153 (0%) [79]**
	3	102 (94%)	154 (10%)	**151 (3%) [42]**	197 (37%)	286 (68%)	140 (7%)	140 (6%)	**148 (0%) [79]**
	4	- (100%)	122 (19%)	105 (80%)	**151 (5%) [59]**	212 (9%)	106 (77%)	110 (64%)	**144 (0%) [78]**
	5	- (100%)	90 (68%)	78 (92%)	110 (13%)	**151 (3%) [68]**	84 (97%)	86 (96%)	**128 (0%) [74]**
	6	108 (96%)	164 (5%)	154 (16%)	207 (35%)	295 (79%)	**151 (2%) [44]**	152 (5%)	**149 (0%) [79]**
	7	122 (96%)	170 (15%)	163 (26%)	217 (51%)	311 (86%)	156 (2%)	**157 (4%) [47]**	**151 (0%) [79]**

E Fit of Estimated RITA Characteristics, Vitros-LS

The method of maximum likelihood, outlined in Section B above, was used to characterise the Vitros-LS, in the South African repeat donor population. Firstly, the mean recency duration, ω, was estimated assuming a known α. Secondly, simultaneous estimation of ω and α was performed. Non-parametric estimation was applied, using only data points with interdonation (ID) intervals larger than T. In the figures below, the observed percentages and expected percentages (obtained by substituting estimated RITA characteristics into (7)) of seroconverters who were recently infected at the first seropositive donations, as a function of ID interval, are compared for $T = 1$ year ($n = 108$) and $T = 2.5$ years ($n = 59$). Subjects with similar ID intervals were grouped together (at least 20 subjects per group), and the observed and expected percentages were plotted against the average ID interval, per group. In Figures 9 and 11, the 95% confidence interval limits for the expected percentages (dotted lines) are based on the 95% confidence interval limits for ω, not taking any uncertainty in α into account. In Figures 10 and 12, the plotted limits for the expected percentages (dotted lines) indicate the minimum and maximum percentages obtained when considering all pairs of values for the RITA characteristics lying within the 95% confidence regions for ω and α.

Figure 9: Agreement between the observed and expected percentages of recently infected seroconverters for the Vitros-LS, in the South African repeat donor population, assuming a known α, for $T = 1$ year

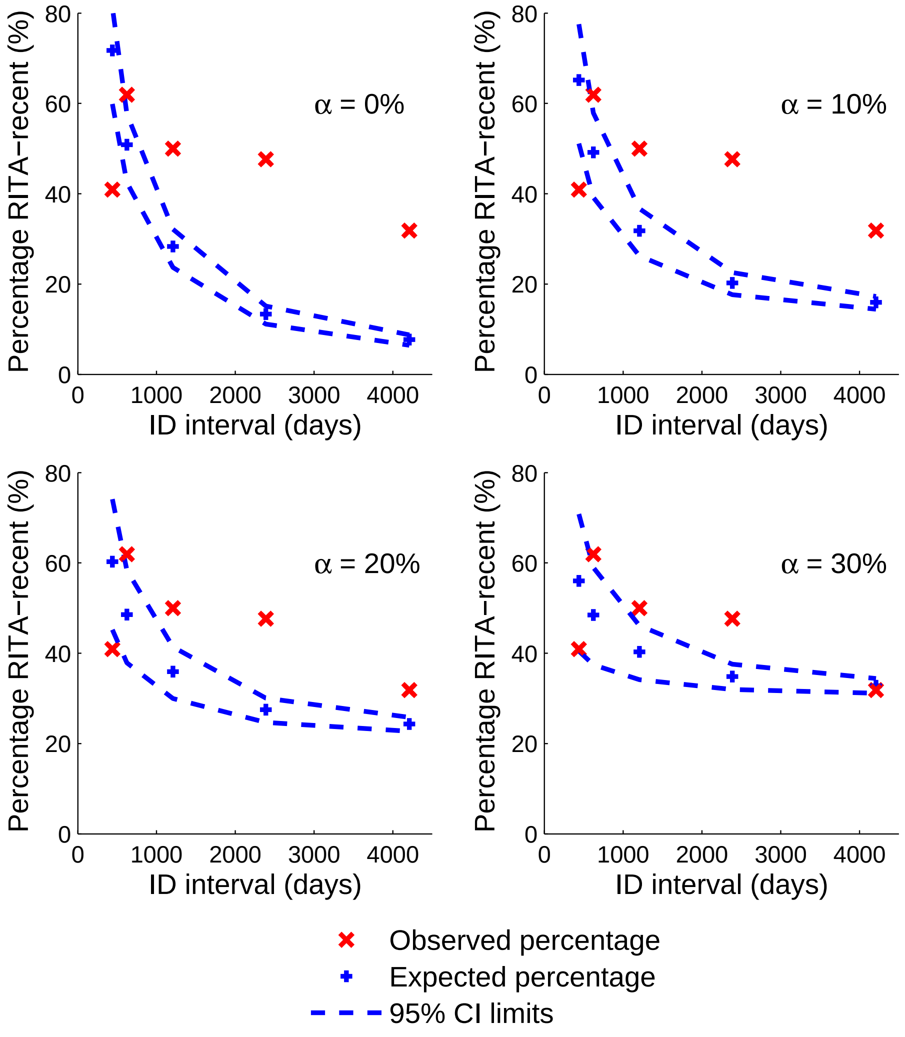

Figure 10: Agreement between the observed and expected percentages of recently infected seroconverters for the Vitros-LS, in the South African repeat donor population, simultaneously estimating ω and α, for $T = 1$ year

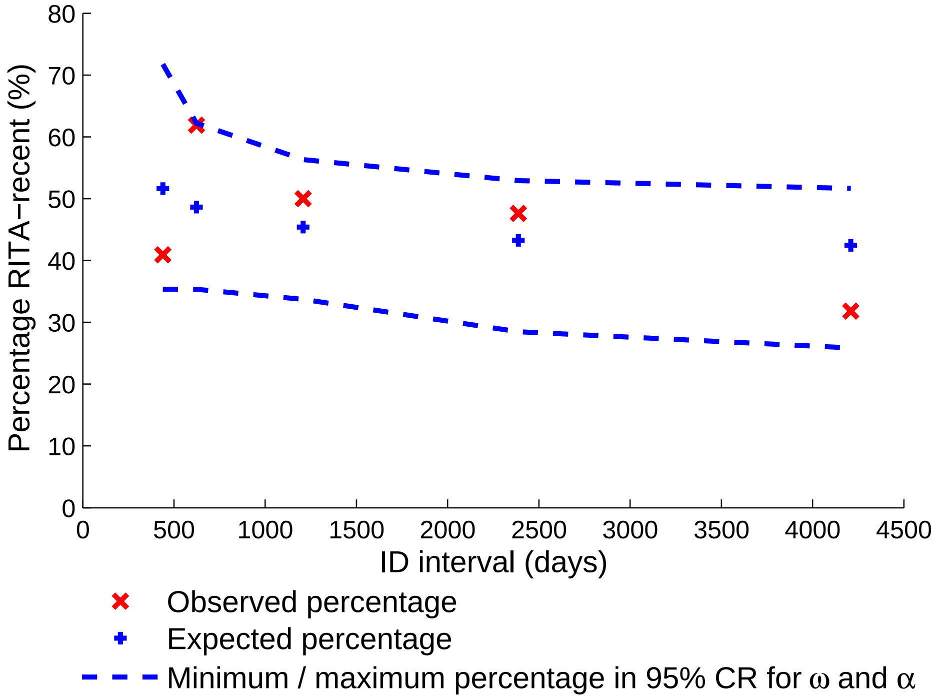

Figure 11: Agreement between the observed and expected percentages of recently infected seroconverters for the Vitros-LS, in the South African repeat donor population, assuming a known α, for $T = 2.5$ years

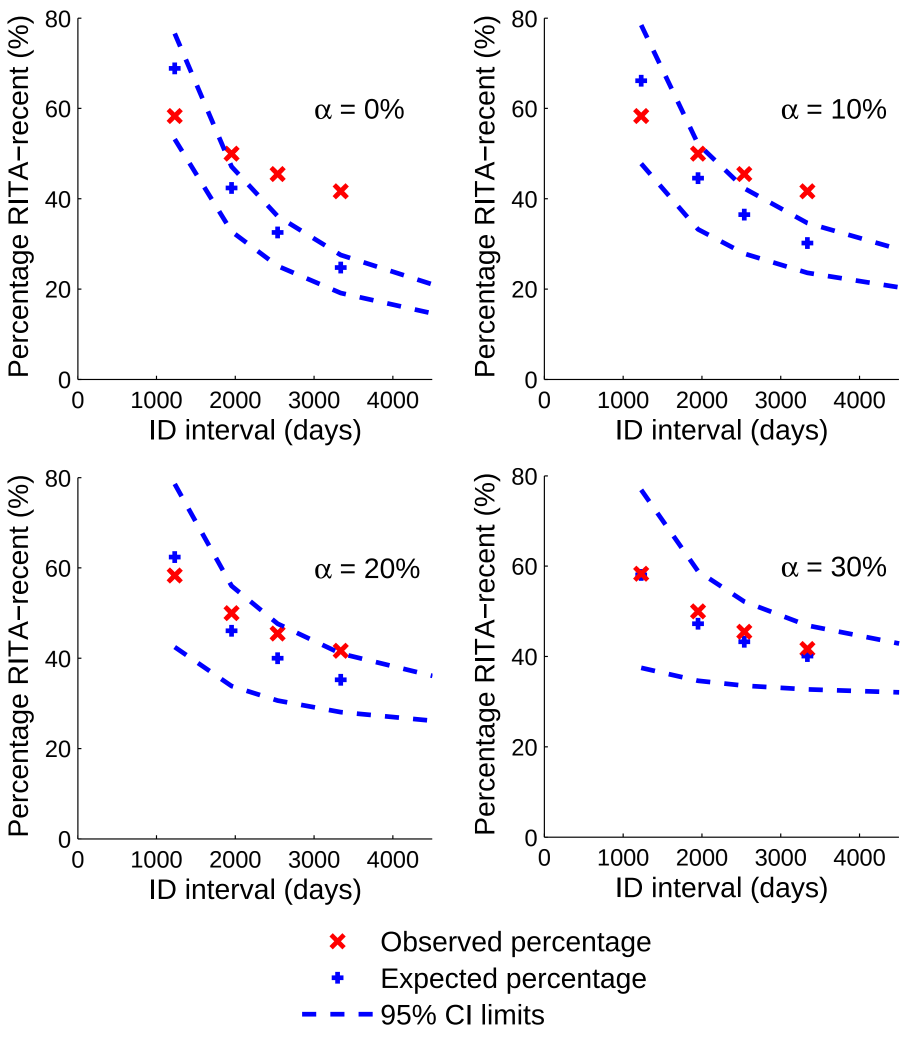

Figure 12: Agreement between the observed and expected percentages of recently infected seroconverters for the Vitros-LS, in the South African repeat donor population, simultaneously estimating ω and α, for $T = 2.5$ years

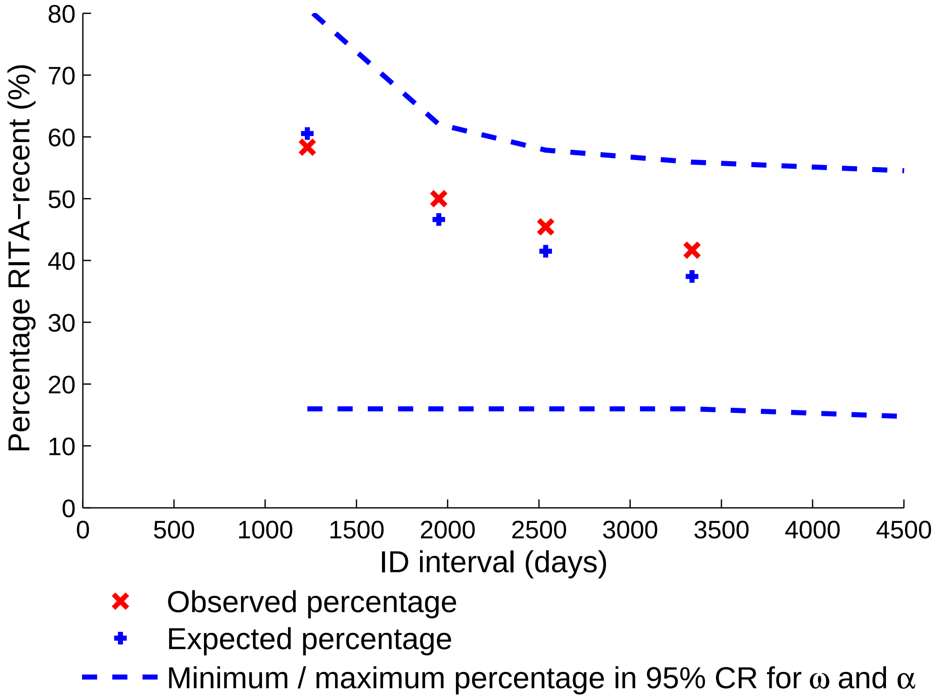

References

1. J S McDougal, B S Parekh, M L Peterson, B M Branson, T Dobbs, M Ackers, and M Gurwith. Comparison of HIV type 1 incidence observed during longitudinal follow-up with incidence estimated by cross-sectional analysis using the BED capture enzyme immunoassay. *AIDS Res Hum Retroviruses*, 22:945–952, 2006.

2. T A McWalter and A Welte. Relating recent infection prevalence to incidence with a sub-population of assay non-progressors. *J Math Biol*, 60:687–710, 2010. Also see poster MOPDB105 at: 5th IAS Conference on HIV Pathogenesis, Treatment and Prevention; 2009; South Africa.

3. R Wang and S W Lagakos. On the use of adjusted cross-sectional estimators of HIV incidence. *J Acquir Immune Defic Syndr*, 52:538–547, 2009.

4. A Welte, T A McWalter, O Laeyendecker, and T B Hallett. Using tests for recent infection to estimate incidence: problems and prospects for HIV. *Euro Surveill*, 15:pii=19589, 2010.

5. D R Cox. *Principles of Statistical Inference.* Cambridge University Press; 2006, New York.

6. J D Hart. *Nonparametric Smoothing and Lack-of-Fit Tests.* Springer-Verlag; 1997, New York.
